# Cancer-related fatigue classification based on heart rate variability signals from wearables

**DOI:** 10.3389/fmed.2023.1103979

**Published:** 2023-04-26

**Authors:** Chi-Huang Shih, Pai-Chien Chou, Jin-Hua Chen, Ting-Ling Chou, Jun-Hung Lai, Chi-Yu Lu, Tsai-Wei Huang

**Affiliations:** ^1^Department of Computer Science and Information Engineering, National Chin-Yi University of Technology, Taichung, Taiwan; ^2^Division of Pulmonary Medicine, Department of Internal Medicine, Taipei Medical University Hospital, Taipei, Taiwan; ^3^Division of Thoracic Medicine, Department of Internal Medicine, School of Medicine, Taipei Medical University, Taipei, Taiwan; ^4^Graduate Institute of Data Science, Taipei Medical University, Taipei, Taiwan; ^5^School of Nursing, College of Nursing, Taipei Medical University, Taipei, Taiwan; ^6^Division of Gastroenterology and Hepatology, Department of Internal Medicine, Erlin Christian Hospital, Changhua, Taiwan; ^7^Department of Nursing, Wan Fang Hospital, Taipei Medical University, Taipei, Taiwan; ^8^Cochrane Taiwan, Taipei Medical University, Taipei, Taiwan; ^9^Research Center in Nursing Clinical Practice, Wan Fang Hospital, Taipei Medical University, Taipei, Taiwan

**Keywords:** cancer-related fatigue, heart rate variability, LF/HF ratio, photoplethysmography, wearables

## Abstract

**Background:**

Cancer-related fatigue (CRF) is the most distressing side effect in cancer patients and affects the survival rate. However, most patients do not report their fatigue level. This study is aimed to develop an objective CRF assessment method based on heart rate variability (HRV).

**Methods:**

In this study, patients with lung cancer who received chemotherapy or target therapy were enrolled. Patients wore wearable devices with photoplethysmography that regularly recorded HRV parameters for seven consecutive days and completed the Brief Fatigue Inventory (BFI) questionnaire. The collected parameters were divided into the active and sleep phase parameters to allow tracking of fatigue variation. Statistical analysis was used to identify correlations between fatigue scores and HRV parameters.

**Findings:**

In this study, 60 patients with lung cancer were enrolled. The HRV parameters including the low-frequency/high-frequency (LF/HF) ratio and the LF/HF disorder ratio in the active phase and the sleep phase were extracted. A linear classifier with HRV-based cutoff points achieved correct classification rates of 73 and 88% for mild and moderate fatigue levels, respectively.

**Conclusion:**

Fatigue was effectively identified, and the data were effectively classified using a 24-h HRV device. This objective fatigue monitoring method may enable clinicians to effectively handle fatigue problems.

## 1. Introduction

Cancer-related fatigue (CRF), defined as a persistent, painful sense of physical, emotional, and cognitive exhaustion related to cancer, is a multidimensional phenomenon that develops over time and can severely affect mobility and be psychologically impaired (1, 2). Moderate-to-severe CRF affects up to 90% of patients who undergo chemotherapy, with approximately 30–40% experiencing fatigue for years after treatment completion (1). CRF is generally identified by active reporting by cancer patients or by active assessment by healthcare professionals using scales or questionnaires. However, patients with CRF can gradually become accustomed to an impaired physical condition and may consider discomfort to be normal (1). Therefore, CRF is often underestimated and untreated. Furthermore, most of the methods used to measure CRF are relatively subjective. Therefore, developing objective tools that encourage effective communication between patients, caregivers, and healthcare providers regarding early reports can improve the efficiency of fatigue management.

Most studies have subjectively evaluated the severity of CRF through questions. The Brief Fatigue Inventory (BFI) is one of the most used means of rapidly assessing CRF in patients with cancer (3). The BFI is a simple, self-administered, and easily scored fatigue scale. The psychometric properties of the BFI have been established in many countries, and the scale has good reliability and validity (4). According to a multivariate analysis of variance between BFI scores and fatigue interference items, three levels of fatigue severity, namely, mild, moderate, and severe, can be identified using cutoff point BFI scores of 1–3, 4–6, and 7–10, respectively (5, 6). In addition, hematological variables, including albumin and hemoglobin levels, were highly correlated with BFI scores in patients with cancer. Although fatigue levels can be effectively monitored using CRF assessment tools, such as BFI, without invasive blood tests, such tools require healthcare professionals to perform active assessments to understand the trajectory of fatigue.

Heart rate variability (HRV) is not new, but there are many innovative applications (7), especially in health and wellbeing (8). HRV is typically classified into two categories: time domain and frequency domain. Frequency-domain measurements of HRV are based on the analysis of the RR intervals using signal processing and frequency techniques. The RR interval denotes the duration between every identified heartbeat, ascertained from the peak (R) to the peak (R) on the QRS complex. HRV may be associated with fatigue and its corresponding effects, such as poor sleep quality. The measurements in the frequency domain of HRV include high frequencies (HF), between 0.14 and 0.4 Hz, and low frequencies (LF), between 0.05 and 0.15 Hz. The LF to HF ratio (LF/HF) represents the relative activity between the sympathetic nervous system and the parasympathetic nervous system under controlled conditions (1). HRV has been used to measure fatigue in numerous situations and in various disease groups (9), and has been used to identify states of wakefulness and fatigue (10). Although further verification is needed, evidence suggests that higher levels of norepinephrine in the brain may be the cause of these symptoms (11). In states of non-rapid eye movement (non-REM), the LF/HF ratio gradually decreases as sleep deepens (10, 12). The LH/HF ratio reflects sleep activity, and higher levels of fatigue can lead to higher frequencies of rest during the day, which can affect sleep quality at night. Additionally, CRF can be associated with impaired autonomic nervous system (ANS) function; the neurotoxic effects of cancer itself, metastasis, surgery, radiation therapy, and chemotherapy can lead to direct and indirect structural damage to the ANS (13, 14). Several studies and case reports have characterized cancer and its treatment as causes of autonomic dysregulation, and abnormal HR recovery was associated with shorter survival times (14, 15).

Studies have shown that wrist-based photoplethysmography (PPG) device data can be consistent with RR intervals from electrocardiography (ECG) devices for heart rate variability analysis (16). In a pilot study (17), we sampled HRV signals from 12 patients with lung cancer using a wearable-based PPG device measurement system. Data were collected using HRV measurements completed once every hour for 24 h for seven consecutive days. Regression analysis of the data revealed that the parameters of the LF/HF ratio that were labeled as active and sleep phases were linearly correlated with the BFI scores. That is, patients with a higher LF/HF ratio in the sleep phase or a lower LF/HF ratio in the active phase had higher fatigue scores.

In this study, as a continuing work of the previous study, objective CRF assessment criteria based on HRV signals measured through wearable devices with PPG sensors were developed. Therefore, the characteristics of the data, including the correlations in the characteristics and cutoff points between mild and moderate CRF categories, from 60 patients with lung cancer, were identified. Based on the cutoff points, the linear classification models were developed with a weighted voting strategy to effectively differentiate the moderate CRF category from the mild one.

## 2. Materials and methods

### 2.1. HRV measurement

In this study, a PPG smartband developed by ViPCare, Gadgle Creative Tech, Taiwan was used to collect PPG signals from the participants. Table 1 presents the validity results by comparing the PPG smartband with a reference ECG device (QHRV, Medeia) in terms of HRV parameters in the time domain and frequency domain. 3-min resting HRV recordings were collected via both smartband and ECG device with a three-lead arrangement in a sitting position. The signal sampling rate was set to 100 and 200 Hz for the smartband and the ECG device, respectively. Three male healthy participants, aged between 20 and 40 years, were involved in the experiments and each of them conducted the HRV recording experiment three times. Time domain parameters included heart rate (HR) in beats/min (BPM), the mean value in ms, and the standard deviation of the NN intervals (SDNN) in ms. The NN interval data is essentially synonymous with RR interval data, albeit with an additional filtering process designed to eliminate artifacts and noise that may render certain RR intervals unreliable. The mean value and SDNN corresponded to the key statistical characteristics of the beat-to-beat intervals measured from the ECG or PPG signals. On the contrary, the frequency domain parameters consisted of LF, HF, and LF/HF ratio. As shown in Table 1, the mean value results of the PPG signals were highly correlated with those of the ECG signals while the PPG-based HRV data typically had higher SD values. According to the accuracy values computed from the HRV parameters based on PPG and ECG, each PPG-based HRV parameter can reach a higher accuracy value than 95%.

**TABLE 1:** Accuracy comparison between ECG and PPG signals in terms of time domain and frequency domain parameters.

		Time domain			Frequency domain		
		HR (BPM)	Mean (ms)	SDNN (ms)	LF (ms^2^)	HF (ms^2^)	LF/HF
ECG	Avg.	91 11.09	657.66 92.20	106.62 19.43	717.91 141.34	714.23 114.15	1.01 0.06
	SD						
PPG	Avg.	93.43 19.65	661.33 146.36	103.22 15.90	749.03 180.45	727.72 141.92	1.03 0.13
	SD						
ACC (%)		97.33	99.44	96.81	95.67	98.11	98.02

ACC, accuracy; avg., average; BPM, beats per minute; ECG, electrocardiography; HR, heart rate; ms, milliseconds; PPG, photoplethysmography; SDNN, standard deviation of the NN intervals.

With the validated PPG smartband, a wearable measurement system periodically triggers hourly HRV measurements over a 24-h period for 7 days to allow observation of long-term CRF trends. Based on other studies (18), the system used in this study was designed to obtain 3-min HRV measurements to obtain the heart rate and two frequency domain parameters (i.e., LF and HF), which corresponded to the sympathetic and parasympathetic activities of the participants.

**FIGURE 1:**
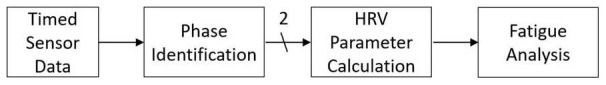
Cancer-related fatigue analysis procedure.

The procedure to analyze the CRF is presented in Figure 1. The timed sensor data include HRV parameters with their timestamps. The HRV parameters can be classified into different phases according to their timestamps. We considered two phases, the active and sleep phases, because these phases correspond to daytime and nighttime measurements, respectively. Since these phases could vary from day to day, an investigator recorded daily activities during the research period. Furthermore, the HRV parameters for each stage were statistically calculated. Based on the data collected from wearable devices, three HRV metrics were selected, namely, the average LF/HF ratio in the sleep phase, the LF/HF disorder ratio in the sleep phase, and the LF/HF disorder ratio in the active phase, to monitor the CRF. For a participant P, the LF/HF disorder ratio in the sleep phase is defined as follows:


(1) $$L\,H\,D_{P}^{S\,l\,e\,e\,p}=\frac{N\,u\,m\,b\,e\,r\,o\,f\,\frac{L\,F}{H\,F}>1\,i\,n\,t\,h\,e\,s\,l\,e\,e\,p\,p\,h\,a\,s\,e}{T\,o\,t\,a\,l\,n\,u\,m\,b\,e\,r\,o\,f\,\frac{L\,F}{H\,F}\,m\,e\,a\,s\,u\,r\,e\,m\,e\,n\,t\,s\,i\,n\,t\,h\,e\,s\,l\,e\,e\,p\,p\,h\,a\,s\,e}$$

CRF may be due to symptom discomfort or poor nighttime sleep, and the LF/HF disorder ratio in the sleep phase was used to track the association between sleep quality and CRF Higher $L\,H\,D_{p}^{S\,l\,e\,e\,p}$ (LHDS) value indicating the target participant experienced a shorter duration of deep sleep. The LF/HF disorder ratio in the active phase of a participant P is defined as follows:


(2) $$L\,H\,D_{P}^{A\,c\,t}=\frac{N\,u\,m\,b\,e\,r\,o\,f\,\frac{L\,F}{H\,F}<1\,i\,n\,t\,h\,e\,a\,c\,t\,i\,v\,e\,p\,h\,a\,s\,e}{T\,o\,t\,a\,l\,n\,u\,m\,b\,e\,r\,o\,f\,\frac{L\,F}{H\,F}\,m\,e\,a\,s\,u\,r\,e\,m\,e\,n\,t\,s\,i\,n\,t\,h\,e\,a\,c\,t\,i\,v\,e\,p\,h\,a\,s\,e}$$

CRF may be due to daytime fatigue or inactivity, and the LF/HF disorder ratio in the active phase was primarily used to track the contribution of the daytime rest to the fatigue condition, Higher $L\,H\,D_{P}^{A\,c\,t}$ (LHDA) value representing excessive daytime sleepiness observed in the daytime.

### 2.2. Participants

The study was approved by the Joint Institutional Review Board of Taipei Medical University (File No. N201910036). The study was conducted using a convenient sampling method in the thoracic clinic and thoracic ward of Taipei Medical University Hospital. The researchers described the study to the patients with lung cancer who received chemotherapy or targeted chemotherapy and obtained informed consent from them. The inclusion criteria were (1) age ≥20 years and (2) the ability to wear the PPG watch device. Patients with weak consciousness and who could not respond to the questionnaires were excluded. A total of 60 patients were included. The data collected included demographic data from patients, LF/HF ratios, and BFI scores. The patients wore the wearable devices for seven consecutive days, enabling HRV measurements once every hour.

### 2.3. Sampling procedures

The aims and objectives of the study were explained to the participants, and written informed consent was obtained before the investigation. The participants were asked to complete the BFI, Taiwanese version (19) and started to wear the PPG watch devices immediately after completing the questionnaire. The participants wore the PPG watch device continuously for 7 days, apart from when they bathed. The participants were reminded through telephone to ensure that they wore the device throughout the research period. After the full 7 days of data were collected, participants returned the devices at their next visit or by mail.

### 2.4. Statistical analysis

IBM SPSS software (version 23.0, IBM Corporation) was used for statistical analyzes. Descriptive statistics, including percentages, means, and standard deviations (SD), were used to present the general characteristics of the data. The three classifications of fatigue are mild (1–3 points), moderate (4–7 points), or severe (8–10 points), and this cutoff point can be used to quickly screen for fatigue (6). With BFI = 4, the cutoff line for the three indicators of heart rate variability can be divided. The independent sample *t*-test was used to estimate the contributions of BFI to three HRV metrics, namely, LHDA, LHDS, and the average LF/HF ratio in the sleep phase for a participant (LH_*p*_, LHP). Consequently, we analyzed the results of the *t*-test to identify cutoff points between the BFI-measured CRF categories for HRV metrics. The weighted majority algorithm (WMA) is a method of training a linear classifier (20). The algorithm adjusts the weights of the input features to identify a linear combination of them that can correctly categorize the examples given a set of input/output examples. We further developed the HRV-based linear classification models with cutoff points were further developed to classify different levels of fatigue.

## 3. Results

### 3.1. Patient characteristics

The descriptive statistics of the 60 patients are summarized in Table 2. The mean age of the patients was 65.9 (SD 9.5) years and most of them were women. Most of the patients had a primary diagnosis of lung adenocarcinoma (90%) and were at an advanced stage of cancer (IV; 80%). Target therapy with epidermal growth factor receptor–tyrosine kinase inhibitors (EGFR–TKI) was used most frequently (65%). In addition, 51.7% of patients were treated with cardiovascular drugs. Most of the patients did not receive hypnotics (85%), and most experienced some form of discomfort, but were almost fully ambulant during the research period (Eastern Cooperative Oncology Group Performance Status = 1).

**TABLE 2:** Descriptive statistics (*N* = 60).

Variable	*n*	%	Mean
Age (year)			65.9 ± 9.5
Sex			
Female	34	56.7	
Male	26	43.3	
Primary diagnosis			
Lung adenocarcinoma	54	90.0	
Lung squamous or small cell carcinoma	6	10.0	
**Stage**			
I + II	6	10.0	
III	6	10.0	
IV	48	80.0	
**Treatment**			
Target (EGFR–TKI)	39	65	
Chemotherapy	21	35	
**Cardiovascular drugs**			
No	31	51.7	
Yes	29	48.3	
**Hypnotics**			
No	51	85.0	
Yes	9	15.0	
**ECOG**			
0	26	43.3	
1	30	50.0	
2	4	6.7	

ECOG, Eastern cooperative oncology group performance status; EGFR–TKIs, epidermal growth factor receptor–tyrosine kinase inhibitors.

### 3.2. Correlation between BFI and HRV

**FIGURE 2:**
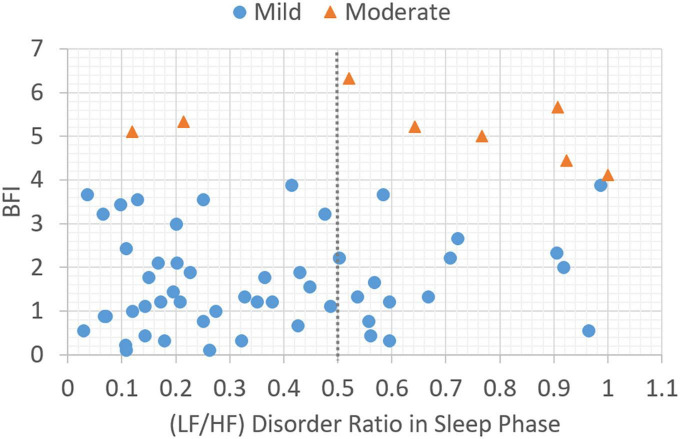
Plot of data distribution between BFI and (LF/HF) disorder ratio in sleep phase; circles represent cases with mild fatigue (BFI < 4), and triangles represent cases with moderate fatigue (BFI ≥ 4). The dotted line illustrates a boundary line with cutoff point, LHDS = 0.5.

**FIGURE 3:**
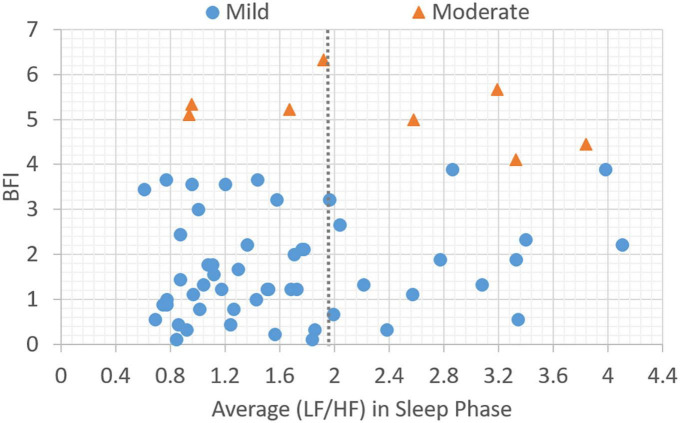
Plot of data distribution between BFI and average (LF/HF) ratio in sleep phase; circles represent cases with mild fatigue (BFI < 4), and triangles represent cases with moderate fatigue (BFI ≥ 4). The dotted line illustrates a boundary line with cutoff point, *LH*_*P*_ = 1.9.

**FIGURE 4:**
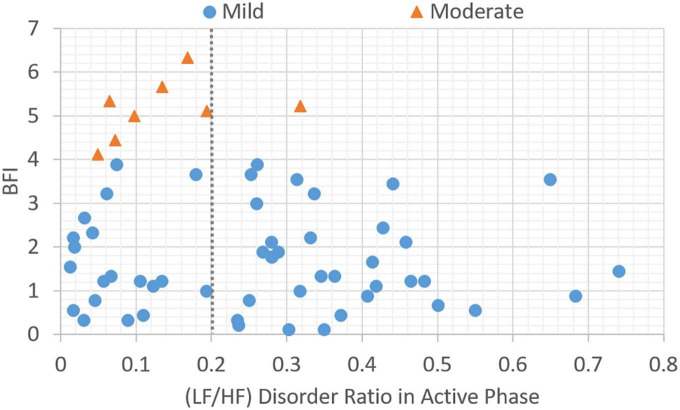
Plot of data distribution between BFI and (LF/HF) disorder ratio in active phase; circles represent cases with mild fatigue (BFI < 4), and triangles represent cases with moderate fatigue (BFI ≥ 4). The dotted line illustrates a boundary line with cutoff point, LHDA = 0.2.

In this section, the association between subjective BFI scores and objective HRV data is analyzed. Patients with a subjective fatigue of four or higher will be classified as moderately fatigued for their corresponding heart rate variability values. Figure 2 presents data distribution of LHDS classified by BFI boundary line. As shown in Figure 2, the moderate CRF category generally has a larger value of LHDS, while the cases belonging to mild category are grouped in a range of smaller values of LHDS. Figures 3–4 show the data distribution for other two HRV metrics, namely, average LF/HF ratio in the sleep phase for a participant *P* and LHDA, respectively.

Based on the data presented in Figures 2–4, the independent samples’ *t*-test was conducted to identify the correlation between HRV metrics and BFI categories. Table 3 lists the *p*-values for the three HRV parameters, with BFI values considered as classification boundaries. In the case of BFI = 4, the *p*-value for LHDS was less than a conservative level of significance (0.05) and the *p*-values for LHP and LHDA were less than a moderate level of significance (0.01). This indicates that the HRV dataset with BFI ≥4 could be differentiated from that with BFI <4. The general classifications of the CRF determined by multivariate factors analysis are mild (BFI = 1–3) and moderate (BFI = 4–6) (5). Therefore, the HRV data allowed for a mild and moderate CRF classification like that for the gold standard. Table 4 presents the mean and standard deviation (SD) of HRV metrics for mild and moderate categories as a boundary line of BFI = 4 is considered. For both LHDS and LHP *LH*_*P*_, patients with moderate CRF have a higher mean value than patients with mild CRF. On the contrary, mild CRF have a larger mean value of LHDA than moderate CRF ones.

**TABLE 3:** The *p*-values for the three HRV parameters under various BFI boundaries.

HRV metrics BFI	${\mathrm{LHD}}_{P}^{\mathrm{Sleep}}$	*LH*_*P*_	${\mathrm{LHD}}_{P}^{\mathrm{Act}}$
2	0.071*	0.062*	0.220
3	0.195	0.221	0.364
4	0.008**	0.065*	0.055*
5	0.469	0.976	0.329

*p < 0.10; **p < 0.05.

**TABLE 4:** Mean and standard deviation for three HRV parameters in a case of BFI = 4.

HRV metrics	${\mathrm{LHD}}_{P}^{\mathrm{Sleep}}$		*LH*_*P*_		${\mathrm{LHD}}_{P}^{\mathrm{Act}}$	
Category	Mean	Mod.	Mean	Mod.	Mean	Mod.
Mean	0.36	0.64	1.65	2.30	0.27	0.14
SD	0.07	0.11	0.77	1.21	0.03	0.01

### 3.3. Cutoff point analysis for HRV metric

As the statistical results reported in Table 3 indicate that HRV metrics can classify mild and moderate CRF categories; therefore, it is essential to identify the cutoff point between CRF categories for each of the three HRV metrics. When searching for the cutoff point, the independent sample *t*-test was performed on two sets of BFI divided by a predetermined value of HRV metrics. Although the *p*-value was statistically lower than a specified point (e.g., 0.05), the associated HRV value can be regarded as a potential candidate for the cutoff point. Additionally, the accuracy of the classification was considered to examine the effectiveness of the classification. This process was repeated for all individual values of HRV metrics. Here, *N*_*i*_ is denoted as the amount of data belonging to the category, *i*, and $N\,P_{i}^{j}$ is denoted as the number of data classified as the category, *i*, by HRV metric *j*. In this study, the category set is {mild, mod}, where “mod” stands for “moderate,” and the HRV metrics set is {LHDS, *LH*_*P*_, LHDA}. Furthermore, the classification accuracy for HRV metrics, *j*, can be calculated.


(3) $$A_{i}^{j}=\frac{N\,P_{i}^{j}}{N_{i}}$$

Based on the above equation, we further define a classification coefficient (CC) to evaluate the overall classification performance for two CRF categories.


(4) $$CC\left(j,m\right)=[\left(\frac{A_{m\,i\,l\,d}^{j}\,\left(m\right)+A_{m\,o\,d}^{j}\,\left(m\right)}{2}\right)+$$

$$\left(1-|A_{m\,i\,l\,d}^{j}\left(m\right)-A_{m\,o\,d}^{j}\left(m\right)|\right)]\times \frac{1}{2}$$

Where *m* is the predetermined value of HRV metrics. In equation (4), the first term calculates an accuracy average of two categories, while the second term estimates the magnitude of the accuracy difference between categories. These two items are then added and normalized by a value of 2. Consequently, the value of CC ranges from 0 to 1. A larger CC indicates that the sum of the classification accuracies of the two classes is larger and the difference is smaller. For the given HRV metrics, *j*, a cutoff point candidate, *m*, can be obtained by maximizing the classification coefficient:


(5) a⁢r⁢g⁢m⁢a⁢x⁢C⁢C⁢(j,m)

**FIGURE 5:**
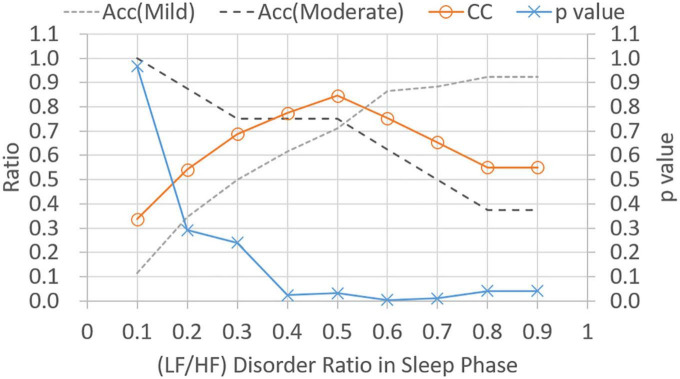
Classification ratio and *p*-value obtained from *t*-test between mild and moderate categories for varied (LF/HF) disorder ratio in sleep phase. “Acc” stands for classification accuracy.

**FIGURE 6:**
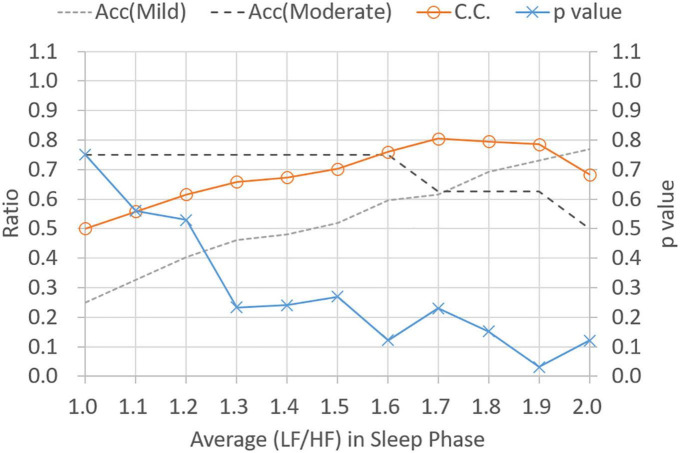
Classification ratio and *p*-value obtained from *t*-test between mild and moderate categories for varied average (LF/HF) ratio in sleeping phase. “Acc” stands for classification accuracy.

**FIGURE 7:**
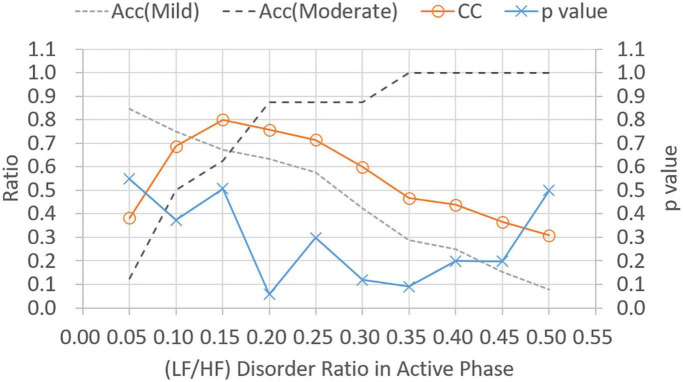
Classification ratio and *p*-value obtained from *t*-test between mild and moderate categories for varied (LF/HF) disorder ratio in the active phase. “Acc” stands for classification accuracy.

Figure 5 shows *p*-values and CC results for varied LF/HF disorder ratios in sleep phase. The LHDS values under consideration ranged from 0.1 to 0.9 in a step of 0.1. As shown in Figure 5, it is observed that (1) *p*-values are below 0.05 for LHDS ≥0.4; (2) as the value of LHDS increases, the classification accuracy of moderate category decreases and that of mild category increases; and (3) the CC curve shows a peak value at LHDS = 0.5. According to the observations discussed in the items (1) and (3) of this list, the cutoff point of LHDS between mild and moderate CRF categories is 0.5. Figure 6 presents the *p*-value and CC results for varied average LF/HF in sleep phase. In Figure 6, the maximum CC value occurs at LHP LH_*p*_ = 1.7, and the slightly lower values attained as the value of *LH*_*P*_ *LH*_*P*_ are 1.8 and 1.9. Since only the *p*-value at *LH*_*P*_ *LH*_*P*_ = 1.9 is below 0.05, the cutoff point of *LH*_*P*_*LH*_*P*_ is 1.9 to achieve low *p*-value and high classification coefficient. Figure 7 presents the *p*-value and CC results for varied LF/HF disorder ratios in the active phase. Based on the observations like those shown in Figure 6, the cutoff point of LHDA is 0.2.

### 3.4. Classification model selection

Although the HRV metrics for the CRF classification are determined in Section “3.3. Cutoff point analysis for HRV metric,” the problem persists to select the best classification model built using a combination of HRV metrics. In this paper, the linear classification model is considered to evaluate the CRF classification performance. For each of the three HRV metrics, the linear models are given as follows:


(6) $$\hat{G}=\left\{ \begin{array}{c} M\,o\,d\,e\,r\,a\,t\,e,i\,f\,L\,H\,D_{P}^{S\,l\,e\,e\,p}\geq 0.5,\\ M\,i\,l\,d,L\,H\,D_{P}^{S\,l\,e\,e\,p}<0.5. \end{array}\right.$$

(7) $$\hat{G}=\left\{ \begin{array}{c} M\,o\,d\,e\,r\,a\,t\,e,i\,f\,L\,H_{P}\geq 1.9,\\ M\,i\,l\,d,L\,H_{P}<1.9. \end{array}\right.$$

(8) $$\hat{G}=\left\{ \begin{array}{c} M\,o\,d\,e\,r\,a\,t\,e,i\,f\,L\,H\,D_{P}^{a\,c\,t}\geq 0.2,\\ M\,i\,l\,d,L\,H\,D_{P}^{a\,c\,t}<0.2. \end{array}\right.$$

The classification results of linear models expressed in equations (6–8) can be observed in Figures 2–4, respectively. As shown in Figures 2–4, the category boundary defined by the HRV-based cutoff point is depicted as a dotted line to classify the mild and moderate CRF categories. According to equations (6–8), the classification result of each model is associated with a binary variable, $\hat{C_{i}^{j}}$. The binary variable, $\hat{C_{i}^{j},}$ is set to 1 while the test case is classified as category, *i*, for HRV metrics, *j*, and set to 0 otherwise. In addition to the linear classification models based on single HRV metrics, it is possible to derive a model with three HRV metrics to enhance the robustness of classification. For a combination of three single-metric methods, the linear model based on the weighted voting strategy can be expressed as follows:


(9) $$\hat{G}=i,i\,f\,\sum_{j}\left(w_{j}\times \hat{C_{i}^{J}}\right)>\frac{\sum_{j}w_{j}}{s}$$

where *S* is the number of HRV metrics and *w*_*j*_ stands for the weight of HRV metrics, *j*. Note that since all weights are set to 1, the model expressed in equation (9) employs majority voting to predict a classification decision. That is, model shown in equation (9) predicts category, *i*, while more than one model among those shown in equations (6–8) classify the target case as category, *i*. In model expressed in equation (9), a higher weight indicates a higher priority for the corresponding HRV metrics. From all combinations of three weights (i.e., 3! = 6), the best configuration to achieve the highest classification accuracies for both categories is given as follows:


(10) $$\hat{G}=i,i\,f\,\sum_{j}\left(w_{j}\times \hat{C_{i}^{J}}\right)>\frac{\sum_{j}w_{j}}{S}$$

where *S* is the number of HRV metrics and *w*_*j*_ stands for the weight of HRV metrics, *j*. Note that since all weights are set to 1, model shown in equation (9) employs majority voting to predict a classification decision. That is, model expressed in equation (9) predicts category, *i*, while more than one model among those expressed in equations (6–8) classify the target case as category, *i*. In model provided in equation (9), a higher weight indicates a higher priority for the corresponding HRV metrics. From all combinations of three weights (i.e., 3! = 6), the best configuration to achieve the highest classification accuracies for both categories is {$w_{LHD_{P}^{Act}}$, $w_{LHD_{P}^{sleep}}$, $w_{LH_{P}}$} in the decreased priority order.

Table 5 gives the classification performance comparison between the models provided in equations (6–9). For models based on single HRV metrics, the linear model expressed in equation (7) can achieve the highest accuracy (0.73) for the mild CRF category, and the model shown in equation (8) has the highest accuracy (0.88) in the moderate category. On the contrary, model shown in equation (6) achieves the classification accuracies beyond 0.7 for both categories and has the highest CC value of 0.85. Consequently, model expressed in equation (6) can be selected as a good single-metrics classification model due to its performance balance between two categories. For model shown in equations (9), the weighted voting of three single-metric models outperforms all single-metrics models expressed in equations (6–8) in terms of classification accuracy. The values of weight set {$w_{LHD_{P}^{Act}}$, $w_{LHD_{P}^{sleep}}$, $w_{LH_{P}}$} are currently configured as {3, 2, 1}. To conclude, three HRV metrics with their respective cutoff points are effective in differentiating moderate CRF category from mild one, while the linear model based on weighted voting can be further constructed to obtain the best CRF classification results.

**TABLE 5:** Classification results for each combination of three HRV metrics.

Metrics (Model)	Accuracy		Classification coefficient
	Mild	Moderate	
${\mathrm{LHD}}_{P}^{\mathrm{Sleep}}$, equation (6)	0.71	0.75	0.85
*LH*_*P*_, equation (7)	0.73	0.63	0.79
${\mathrm{LHD}}_{P}^{\mathrm{Act}}$, equation (8)	0.63	0.88	0.75
${\mathrm{LHD}}_{P}^{\mathrm{Sleep}},{\mathrm{LH}}_{P},{\mathrm{LHD}}_{P}^{\mathrm{Act}}$, equation (9)	0.73	0.88	0.83

## 4. Discussion

An analysis of HRV data for the 60 patients with lung cancer enrolled in this study revealed that the LF/HF in the active phase and in sleep can be classified as indicating mild and moderate fatigue levels with BFI = 4, which supports the current fatigue classification criteria. Furthermore, in this study, using the cutoff point results obtained using the statistical analysis method, a linear classifier to differentiate fatigue into mild and moderate levels is developed. The analytical results revealed a total correct classification rate of 81% and that the two-phase LF/HF parameters can be considered fair indicators to assess the severity of CRF during cancer treatment.

In our previous pilot study, we found a moderate positive correlation between the average LF/HF ratio and the BFI in the sleep phase (ρ = 0.86). In addition, we define the LF/HF disorder ratio in sleep and active phases (17). That is, we reported that HF increased in the fatigued state and LF increased in the awake state (12). The LF/HF ratios are typically approximately 1 in the non-REM state and are even lower in higher non-REM stages. For comparison, the LF/HF ratio increases in REM state (21). During sleep, the LF/HF disorder ratio increases as the BFI increases (17). In this study, we verified the associations between the LF/HF disorder ratio, the average LF/HF ratio, and the BFI.

During sleep, an increase in metabolic rate (22), an increase in cortisol levels (23), an increase in HRV of LF, and a decrease in HF indicate physiological hyperarousal (24). Furthermore, these phenomena may indicate that an individual experiences insomnia and feels tired during subsequent daytime periods. This is associated with activation of the stress system, the sympathetic-adrenomedullary system, and the hypothalamic–pituitary–adrenal (HPA) axis. During nocturnal sleep, normal people experience a decrease in the LF/HF ratio or have an LF/HF ratio lower than 1, reflecting a decrease in sympathetic nerve activity. This enables people to achieve better sleep (25). However, an LF/HF ratio that is not significant in the 24-h activity and sleep stages indicates a disturbance in the circadian balance of the ANS. In this study, the gold standard cutoff point for BFI to classify fatigue levels with a BFI of less than four points into mild and moderate fatigue groups has been used. The average LF/HF ratio for the corresponding sleep stage was 1.9 (i.e., an LF/HF ratio greater than 1.9 was considered moderate fatigue; Figure 3). The LF/HF disorder ratio was 0.5, indicating that patients with an LF/HF disorder ratio greater than 0.5 were in a state of moderate fatigue (Figure 2).

Apart from during nocturnal sleep, the LF/HF ratio can increase or exceed 1 during daytime activity in the normal population. This reflects increased sympathetic activity and people being more active. An LF/HF disorder ratio of less than 1 during the active phase indicates less activity. When the fatigue is greater than 4, a relative active-phase LF/HF disorder ratio greater than 0.2 is considered to indicate moderate fatigue.

Cancer-related fatigue is a common and often long-lasting symptom for many cancer survivors. Patients with cancer and survivors of cancer generally describe their fatigue as more severe, pervasive, and debilitating than their normal fatigue caused by lack of sleep or overexertion (26). A possible cause of increased inflammation in fatigued cancer survivors may be the ANS. The ANS is a key regulator of the immune system, including the inflammatory cytokine network. Inflammation is a local protective response to microbial invasion or injury. Inflammation must be and should be precisely regulated because insufficient or excessive inflammatory responses can lead to morbidity and a shortened lifespan. The nervous system reflexively modulates inflammatory responses as they occur like how it controls heart rate and other vital functions (27). A previous study had demonstrated an association between elevated inflammatory markers and fatigue during and after treatment (28). Generally, activation of sympathetic branches of the ANS can lead to increased inflammation, and activation of parasympathetic branches of the ANS can lead to decreased inflammation (29, 30). However, these effects may be complex and highly correlated (31). The parasympathetic nervous system stimulation (through the vagus nerve) leads to reduced production of proinflammatory cytokines through the release of the neurotransmitter acetylcholine (27, 32). Furthermore, HRV parameters are highly correlated with elevated C-reactive protein, a marker of inflammation (14). Increased activity in the sympathetic branches or decreased activity in the parasympathetic branches may play a role in inflammation and symptoms associated with CRF. A study documented cross-sectional associations between higher parasympathetic activity (measured by HRV) and lower levels of inflammation (33). The importance of the parasympathetic nervous system was also supported by a study that investigated the association between autonomic activity and fatigue in non-cancer samples (34). Research also demonstrated that individuals with chronic fatigue syndrome have a reduced HRV compared to healthy controls (35) like women who report stress-related fatigue (10). Fagundes et al. (36) discovered that lower resting HRV was associated with higher levels of fatigue. Studies have also demonstrated that HRV is significantly lower in patients with cancer than in the healthy population (37) and that decreased HRV was associated with significantly shorter survival in patients with cancer (38). Furthermore, CRF may negatively affect quality of life and indicate shorter survival (39, 40).

Previous clinical measurements of HRV have been obtained from stationary devices in a denoised environment (such as a physiological laboratory with good environmental noise isolation). Hence, only a limited active period of data (usually 5 min onward) can be collected during such measurements. Furthermore, the combined influence of the sympathetic and parasympathetic nerves can be determined through the LF and HF bands (41). In this study, a 24-h HRV collection using a PPG device was conducted to distinguish between active and sleep phases to further distinguish the mixed effects of the sympathetic and parasympathetic nerves. In general, dysregulation of the autonomic nervous system usually refers to a dysregulation or decrease in HRV during the active phase. That is, researchers have reported that increased parasympathetic nerve activity during the active phase indicates a higher level of fatigue.

The 30-s sit-to-stand (30-STS) test is another potential assessment tool that can complement patient fatigue beyond self-report (42). It assesses functional fitness by evaluating the number of repetitions completed and has been used to measure lower extremity endurance in cancer survivors (43). However, the 30-STS cannot be assessed in cancer patients who are unable to stand for measurement. On the contrary, wrist-based PPG tools do not have this limitation.

The main contributions of this study include our use of the statistical analysis method for identification and our application of the classification method to categorize mild and moderate fatigue levels using objective HRV metrics. The LF/HF disorder ratio (LF/HF >1) of the sleep phase and the average LF/HF ratio of the sleep phase were positively associated with subjective BFI results, and the LF/HF disorder ratio (LF/HF <1) of the active phase was negatively associated with BFI. Furthermore, HRV data could be used to divide patients into mild and moderate fatigue levels according to BFI = 4. Therefore, the linear classifier obtained from the combination of cutoff points with respect to three HRV indicators was able to effectively categorize mild and moderate fatigue levels. The wrist-based PPG tool can be used to actively record and track fatigue in cancer patients. In the future, it will be integrated with social media to alert and suggest fatigue levels and suggest patients to plan different exercises depending on the level of fatigue. In addition, it will provide objective information in addition to subjective data during cancer treatments, such as chemotherapy.

## 5. Limitations

This study had three main limitations. First, the sample size for moderate fatigue levels was relatively small, and the absence of severe fatigue cases indicates that more samples are required to strengthen the generalizability of our findings. Second, due to the small sample size, we did not adjust for HRV caused by different treatments. Third, sweating when wearing the PPG device may have caused discomfort for some patients and others may have worried about potentially damaging the device during activities that involved water. Therefore, some data may have been lost if patients removed their PPG watch devices at other times than when bathing. This way of calculating fatigue is an innovative approach. An objective test was created along with the application of the most widely used subjective fatigue scale. The WMA helps clarify and classify mild and moderate fatigue levels. Future research involving all fatigue categories, particularly severe fatigue, is required to investigate the validity of HRV-based fatigue classifications.

## 6. Conclusion

This is the first study to use a linear fatigue classifier (involving the LF/HF ratio, active-phase disorder rate, and sleep phase disorder rate) for objective fatigue classification. This classifier could effectively classify the severity of fatigue in patients with lung cancer, with a cutoff point between mild and moderate levels like that of the BFI scores (i.e., BFI = 4). This indicates that HRV can be used for objective assessments of CRF. We propose that objective fatigue monitoring using HRV is required in addition to subjective assessments of CRF. This is particularly true for the fatigue-related nursing intervention; objective fatigue monitoring is required to understand the effects of the intervention.

## Data availability statement

The original contributions presented in this study are included in the article/supplementary material, further inquiries can be directed to the corresponding author.

## Ethics statement

The studies involving human participants were reviewed and approved by the Joint Institutional Review Board of Taipei Medical University. The patients/participants provided their written informed consent to participate in this study.

## Author contributions

C-HS and T-WH: conceptualization, methodology, data curation, and writing the original draft. P-CC, J-HL, C-YL, and T-LC: visualization and investigation. J-HC, C-HS, and T-WH: statistical analysis and interpretation. T-WH: supervision, conceptualization, methodology, and writing—review and editing. All authors contributed to the article and approved the submitted version.

## References

[1] Bower J. Cancer-related fatigue–mechanisms, risk factors, and treatments. *Nat Rev Clin Oncol.* (2014) 11:597–609. 10.1038/nrclinonc.2014.127 25113839 PMC4664449

[2] Cella D Davis K Breitbart W Curt G. Cancer-related fatigue: prevalence of proposed diagnostic criteria in a United States sample of cancer survivors. *J Clin Oncol.* (2001) 19:3385–91. 10.1200/JCO.2001.19.14.3385 11454886

[3] Davis K Lai J Hahn E Cella D. Conducting routine fatigue assessments for use in clinical oncology practice: patient and provider perspectives. *Support Care Cancer.* (2008) 16:379–86. 10.1007/s00520-007-0317-9 17724621

[4] Gebremariam G Anshabo A Tigeneh W Engidawork E. Validation of the amharic version of the brief fatigue inventory for assessment of cancer-related fatigue in Ethiopian cancer patients. *J Pain Symptom Manage.* (2018) 56:264–72. 10.1016/j.jpainsymman.2018.04.015 29753101

[5] Mendoza T Wang X Cleeland C Morrissey M Johnson B Wendt J The rapid assessment of fatigue severity in cancer patients: use of the Brief Fatigue Inventory. *Cancer.* (1999) 85:1186–96. 10.1002/(sici)1097-0142(19990301)85:5<1186::aid-cncr24<3.0.co;2-n 10091805

[6] Chang Y Lee J Lee C Lee W Lee K Bang S Assessment of clinical relevant fatigue level in cancer. *Support Care Cancer.* (2007) 15:891–6. 10.1007/S00520-007-0219-X 17318593

[7] Laborde S Mosley E Bellenger C Thayer J. Editorial: Horizon 2030: innovative applications of heart rate variability. *Front Neurosci.* (2022) 16:832. 10.3389/FNINS.2022.937086/BIBTEX PMC9205192 35720712

[8] Drury R Porges S Thayer J Ginsberg J. Editorial: heart rate variability, health and well-being: a systems perspective. *Front Public Health.* (2019) 7:323. 10.3389/FPUBH.2019.00323/BIBTEX PMC6848255 31750285

[9] Pereira T Moreira T Almeida P Cunha J Aguiar A. Fine grained stress assessment in ecological conditions. *Proceedings of the 37th Annual international conference of the IEEE engineering in medicine and biology society of the IEEE engineering in medicine and biology society 2015 presented at: EMBC’15; March 31, 2015.* Milano: IEEE (2015).

[10] Olsson E. *Heart rate variability in stress-related fatigue: Adolescent anxiety and depression and its connection to lifestyle.* PhD thesis. Uppsala: Acta University Uppsala (2010).

[11] Assad S Ding F Fu N Xu Y. *Correlating heart rate variability with mental fatigue.* Massachusetts, MA: Worchester Polytechnic Institute (2012).

[12] Vicente J Laguna P Bartra A Bailón R. Drowsiness detection using heart rate variability. *Med Biol Eng Comput.* (2016) 54:927–37. 10.1007/s11517-015-1448-7 26780463

[13] Martínez-Navarro I Sánchez-Gómez J Collado-Boira E Hernando B Panizo N Hernando C. Cardiac damage biomarkers and heart rate variability following a 118-Km mountain race: relationship with performance and recovery. *J Sports Sci Med.* (2019) 18:615–22. 31827345 PMC6873135

[14] Saito I Hitsumoto S Maruyama K Eguchi E Kato T Okamoto A Impact of heart rate variability on C-reactive protein concentrations in Japanese adult nonsmokers: the toon health study. *Atherosclerosis.* (2016) 244:79–85. 10.1016/j.atherosclerosis.2015.10.112 26595902

[15] Meinardi M van Veldhuisen D Gietema J Dolsma WV Boomsma F van den BM Prospective evaluation of early cardiac damage induced by epirubicin-containing adjuvant chemotherapy and locoregional radiotherapy in breast cancer patients. *J Clin Oncol.* (2001) 19:2746–53. 10.1200/JCO.2001.19.10.2746 11352968

[16] Tarniceriu A Harju J Vehkaoja A Parak J Delgado-Gonzalo R Renevey P Detection of beat-to-beat intervals from wrist photoplethysmography in patients with sinus rhythm and atrial fibrillation after surgery. *Proceedings of the 2018 IEEE EMBS International conference on biomedical and health informatics, BHI 2018.* Piscataway, NJ: IEEE (2018). p. 133–6. 10.1109/BHI.2018.8333387

[17] Shih C Chou P Chou T Huang T. Measurement of cancer-related fatigue based on heart rate variability: observational study. *J Med Internet Res.* (2021) 23:e25791. 10.2196/25791 36260384 PMC8406124

[18] Shaffer F Ginsberg J. An overview of heart rate variability metrics and norms. *Front Public Health.* (2017) 5:258. 10.3389/fpubh.2017.00258 29034226 PMC5624990

[19] Lin C Chang A Chen M Cleeland C Mendoza T Wang X. Validation of the Taiwanese version of the Brief Fatigue Inventory. *J Pain Symptom Manage.* (2006) 32:52–9. 10.1016/j.jpainsymman.2005.12.019 16824985

[20] Littlestone N Warmuth M. The weighted majority algorithm. *Inf Comput.* (1994) 108:212–61. 10.1006/inco.1994.1009

[21] Elsenbruch S Harnish M Orr W. Heart rate variability during waking and sleep in healthy males and females. *Sleep.* (1999) 22:1067–71. 10.1093/sleep/22.8.1067 10617167

[22] Bonnet M Arand D. 24-Hour metabolic rate in insomniacs and matched normal sleepers. *Sleep.* (1995) 18:581–8. 10.1093/sleep/18.7.581 8552929

[23] Vgontzas A Bixler E Lin H Prolo P Mastorakos G Vela-Bueno A Chronic insomnia is associated with nyctohemeral activation of the hypothalamic-pituitary-adrenal axis: clinical implications. *J Clin Endocrinol Metab.* (2001) 86:3787–94. 10.1210/jcem.86.8.7778 11502812

[24] Bonnet M Arand D. Heart rate variability in insomniacs and matched normal sleepers. *Psychosom Med.* (1998) 60:610–5. 10.1097/00006842-199809000-00017 9773766

[25] Parati G Castiglioni P di Rienzo M Omboni S Pedotti A Mancia G. Sequential spectral analysis of 24-hour blood pressure and pulse interval in humans. *Hypertension.* (1990) 16:414–21. 10.1161/01.hyp.16.4.414 2210809

[26] Poulson M. Not just tired. *J Clin Oncol.* (2003) 21:112s–3s. 10.1200/JCO.2003.01.191 12743214

[27] Tracey K. The inflammatory reflex. *Nature.* (2002) 420:853–9. 10.1038/nature01321 12490958

[28] Bower J Lamkin D. Inflammation and cancer-related fatigue: mechanisms, contributing factors, and treatment implications. *Brain Behav Immun.* (2013) 30:S48–57. 10.1016/j.bbi.2012.06.011 22776268 PMC3978020

[29] Irwin M Cole S. Reciprocal regulation of the neural and innate immune systems. *Nat Rev Immunol.* (2011) 11:625–32. 10.1038/nri3042 21818124 PMC3597082

[30] Thayer J Sternberg E. Beyond heart rate variability: vagal regulation of allostatic systems. *Ann N Y Acad Sci.* (2006) 1088:361–72. 10.1196/annals.1366.014 17192580

[31] Sanders V Straub R. Norepinephrine, the beta-adrenergic receptor, and immunity. *Brain Behav Immun.* (2002) 16:290–332. 10.1006/brbi.2001.0639 12096881

[32] Tracey K. Reflex control of immunity. *Nat Rev Immunol.* (2009) 9:418–28. 10.1038/nri2566 19461672 PMC4535331

[33] Sajadieh A Nielsen O Rasmussen V Hein H Abedini S Hansen J. Increased heart rate and reduced heart-rate variability are associated with subclinical inflammation in middle-aged and elderly subjects with no apparent heart disease. *Eur Heart J.* (2004) 25:363–70. 10.1016/j.ehj.2003.12.003 15033247

[34] Appelhans B Luecken L. Heart rate variability as an index of regulated emotional responding. *Rev Gen Psychol.* (2006) 10:229–40. 10.1037/1089-2680.10.3.229

[35] Beaumont A Burton A Lemon J Bennett B Lloyd A Vollmer-Conna U. Reduced cardiac vagal modulation impacts on cognitive performance in chronic fatigue syndrome. *PLoS One.* (2012) 7:e49518. 10.1371/journal.pone.0049518 23166694 PMC3498107

[36] Fagundes C Murray D Hwang B Gouin J Thayer J Sollers J III Sympathetic and parasympathetic activity in cancer-related fatigue: more evidence for a physiological substrate in cancer survivors. *Psychoneuroendocrinology.* (2011) 36:1137–47. 10.1016/j.psyneuen.2011.02.005 21388744 PMC3128662

[37] de Couck M Gidron Y. Norms of vagal nerve activity, indexed by heart rate variability, in cancer patients. *Cancer Epidemiol.* (2013) 37:737–41. 10.1016/j.canep.2013.04.016 23725879

[38] Fadul N Strasser F Palmer J Yusuf S Guo Y Li Z The association between autonomic dysfunction and survival in male patients with advanced cancer: a preliminary report. *J Pain Symptom Manage.* (2010) 39:283–90. 10.1016/j.jpainsymman.2009.06.014 20152590

[39] Bower J Ganz P Desmond K Rowland J Meyerowitz B Belin T. Fatigue in breast cancer survivors: occurrence, correlates, and impact on quality of life. *J Clin Oncol.* (2000) 18:743–53. 10.1200/JCO.2000.18.4.743 10673515

[40] Groenvold M Petersen M Idler E Bjorner J Fayers P Mouridsen H. Psychological distress and fatigue predicted recurrence and survival in primary breast cancer patients. *Breast Cancer Res Treat.* (2007) 105:209–19. 10.1007/s10549-006-9447-x 17203386

[41] Holzman J Bridgett D. Heart rate variability indices as bio-markers of top-down self-regulatory mechanisms: a meta-analytic review. *Neurosci Biobehav Rev.* (2017) 74:233–55. 10.1016/j.neubiorev.2016.12.032 28057463

[42] Cuesta-Vargas A Buchan J Pajares B Alba E Roldan-Jiménez C. Cancer-related fatigue stratification system based on patient-reported outcomes and objective outcomes: a cancer-related fatigue ambulatory index. *PLoS One.* (2019) 14:e0215662. 10.1371/JOURNAL.PONE.0215662 31009501 PMC6476532

[43] Galiano-Castillo N Ariza-García A Cantarero-Villanueva I Fernández-Lao C Díaz-Rodríguez L Legerén-Alvarez M Telehealth system (e-CUIDATE) to improve quality of life in breast cancer survivors: rationale and study protocol for a randomized clinical trial. *Trials.* (2013) 14:187. 10.1186/1745-6215-14-187 23799886 PMC3704734

